# Polyester-Based Dendrimer Nanoparticles Combined with Etoposide Have an Improved Cytotoxic and Pro-Oxidant Effect on Human Neuroblastoma Cells

**DOI:** 10.3390/antiox9010050

**Published:** 2020-01-06

**Authors:** Silvana Alfei, Barbara Marengo, Cinzia Domenicotti

**Affiliations:** 1 Department of Pharmacy, University of Genoa, Viale Cembrano, 16148 Genoa, Italy; 2 Department of Experimental Medicine (DIMES), University of Genova, Via Alberti L.B., 16132 Genoa, Italy; barbara.marengo@unige.it

**Keywords:** etoposide, lab-made biodegradable dendrimer, nanoparticle formulation, improved solubility, neuroblastoma cells, synergistic action, protracted release

## Abstract

Etoposide (ETO) is a cytotoxic drug that exerts its effect by increasing reactive oxygen species (ROS) production. Although ETO is widely used, fast metabolism, poor solubility, systemic toxicity, and multi-drug resistance induction all limit its administration dosage and its therapeutic efficiency. In order to address these issues, a biodegradable dendrimer was prepared for entrapping and protecting ETO and for enhancing its solubility and effectiveness. The achieved dendrimer complex with ETO (CPX **5**) showed the typical properties of a well-functioning delivery system, i.e., nanospherical morphology (70 nm), optimal Z-potential (−45 mV), good drug loading (37%), very satisfying entrapment efficiency (53%), and a remarkably improved solubility in biocompatible solvents. In regards to its cytotoxic activity, CPX **5** was tested on neuroblastoma (NB) cells with very promising results. In fact, the dendrimer scaffold and ETO are able to exert per se a cytotoxic and pro-oxidant activity on human NB cells. When CPX **5** is combined with ETO, it shows a synergistic action, slowly releasing the drug over time and significantly improving and protracting bioactivity. On the basis of these findings, the prepared ETO reservoir represents a novel biodegradable and promising device for the delivery of ETO into NB cells.

## 1. Introduction

In order to exert their anticancer action, the currently used chemotherapeutic drugs are administered at high doses that, with long-lasting treatment, induce serious secondary toxic effects in patients. In this context, several strategies have been devised in order to reduce drug toxicity, such as modifying the schemes and the routes of drug administration. Another strategy, currently being studied and which is giving promising results, is the administration of chemotherapeutic drugs entrapped into nanoparticles, which, by sequestering the drug within them and avoiding direct contact with body fluids, reduce drug toxicity and fast metabolism. Among nanoparticles, dendrimers [1] are easily transported in the blood [2,3], show a low polydispersion index [4], allow a greater bioavailability, and improve the pharmacokinetic of the transported drugs thanks to a series of nonpareil properties [4,5,6], including a low viscosity [2,3].

**Figure 1:**
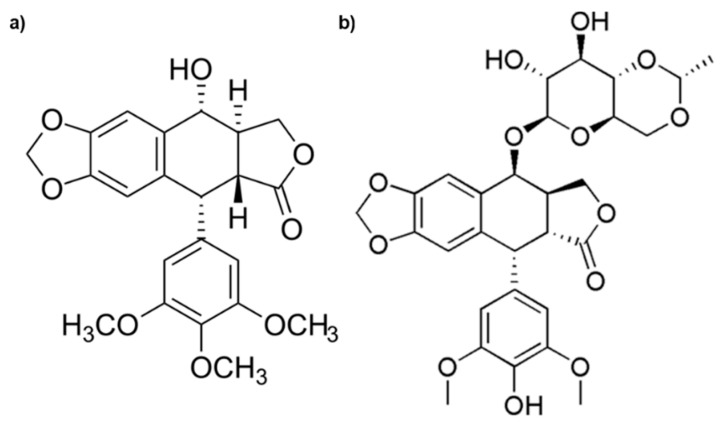
Chemical structures of Podophyllotoxin (**a**) and Etoposide (**b**).

Among the drugs, etoposide (ETO) is widely used to treat severe malignancy, but secondary toxic effects, poor solubility, and the induction of resistance limit its therapeutic efficiency. ETO is derived from Podophyllotoxin (Figure 1), which is the main chemical component of podophyllin, the resin extracted from herbaceous plants known as Podophyllum hexandrum and Podophyllum peltatum [7,8].

In particular, ETO acts by inhibiting topoisomerase II through interactions with the amino acid residues Asp737, Tyr804, Lys738, and Glu784. Alpha and beta toposisomerase II isoforms are differently expressed during the cell cycle. In particular, the alpha isoform, whose level increases during the G2/M phase, is the target of ETO [9]. In fact, ETO is cell cycle-specific and accumulates only in G2/M1 phase cells [10]. Interestingly, ETO is clinically used, in combination with other antineoplastic drugs, for the treatment of patients with neuroblastoma (NB) or many other solid tumors [11,12,13]. Unfortunately, its clinical efficiency is hindered by poor water solubility, metabolic and non-metabolic inactivation, drug resistance, myelosuppression, and poor bioavailability [14,15,16].

**Figure 2:**
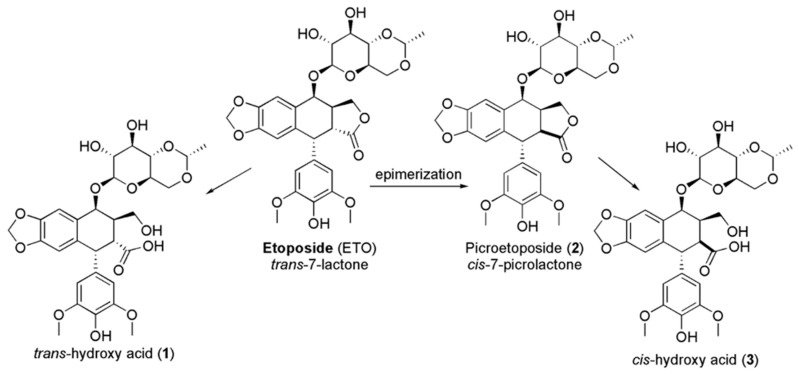
Deactivation of ETO by epimerization.

In addition, high drug concentrations lead to the saturation of the enzyme, with prolonged exposure and great cytotoxicity [17,18,19,20]. ETO encompasses a *trans*-fused 7-lactone ring that, by enzyme action, is promptly metabolized into *trans*-hydroxyl acid (**1**). Moreover, it could epimerize into the *cis*-picro lactone isomer picroetoposide (PETO) (**2**) that, in turn, is hydrolyzed to *cis*-hydroxyl acid (**3**) (Figure 2), metabolites losing in vitro biological activity [14,21,22].

**Figure 3:**
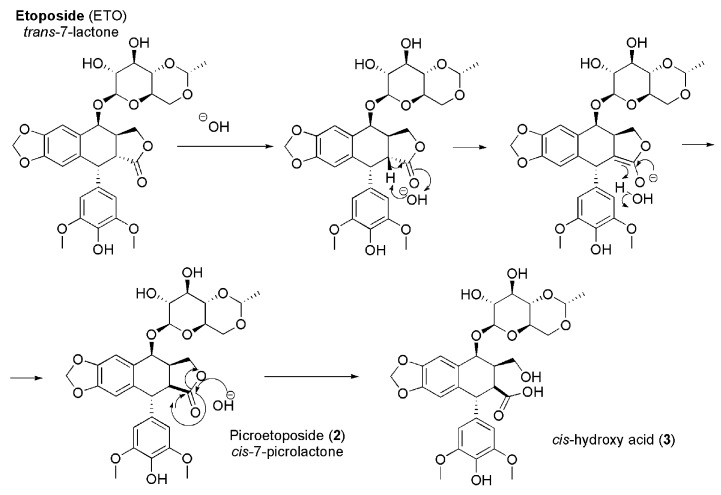
Mechanism of epimerization of ETO.

Furthermore, ETO is also easily inactivated by non-enzymatic epimerization provoked by weak bases. An assumption, concerning the mechanism of the conversion of the *trans* lactone ring to the thermodynamically stable *cis*-lactone, deals with the formation of an enolate at C-2 by proton abstraction, followed by an inversion of the configuration at C-2 [21,23] (Figure 3).

Concerning the inactivating process shown in Figure 3, it is reported that PETO can be selectively produced at pH = 9, while the hydroxyl acid is detectable at pH = 12. At low pH values, the *cis*-hydroxyl acid is reverted to PETO [24].

Some years ago, a series of arylamino derivatives of ETO was prepared in order to achieve compounds more resistant to inactivation and therefore endowed with greater therapeutic efficiency [25].

The study resulted successful, and 4d-(4”-arylamino)-4’-demethylpodophyllotoxin 7-cyclic acetal, 7-cyclic ether, and 7-lactol were obtained. These compounds proved to have the ability to block epimerization and the biological deactivation processes, showing activities equal or superior to ETO.

Despite the promising results, the synthetic path, composed of several steps and the laborious and long purification operations through chromatographic columns, necessary after each step can compromise the actual feasibility of this apparently appealing strategy.

In the last decades, great advances have been made in drug delivery engineered techniques, and, nowadays, the most attractive, fast, and low cost prospect for protecting and delivering ETO, overcoming its limitations, and improving its therapeutic efficiency and clinical applications is represented by the use of proper carrier nano-devices.

Nanoparticles are extensively inspected to prepare multifunctional smart materials [26,27,28,29,30] and are widely employed in various biomedical applications [31,32,33,34,35,36,37,38,39,40,41,42,43,44,45,46,47,48,49].

In this regard, inorganic nanohybrid constructs, consisting of layered double hydroxides (LDHs) intercalated with VP16 (ETO phosphate), have been successfully synthesized and characterized [50]. The prepared nanoparticles showed an average diameter of 62.5 nm, Z-potential of +20.5 mV, drug loading (DL%) of 21.94%, and a profile of sustained release of the drug. In vitro tests showed that VP16-LDH nanoparticles exerted a high cytotoxic effect on cancer cells while being less toxic for healthy cells [50]. However, although the results obtained from the employment of LDHs are promising, further improvements could be important in order to enhance some physicochemical properties, which are pivotal for a well-functioning nanosized drug delivery system, such as Z-potential [51,52,53] and an increase in DL%. Among the strategies adopted, the use of dendrimer offers interesting perspectives [1,2,3,4,5,6]. The commercially available poly (amidoamine) dendrimers (PAMAMs) are well performant in vitro, but they are not advisable for clinical applications because, if not properly modified, are harmful to cell membranes [54]. On the contrary, polyester dendrimer scaffolds show very attractive results because they are highly biodegradable and respectful to physiological membranes [4,55,56].

Starting from this background, the aim of this work was to develop a successful strategy to ameliorate ETO stability and solubility, thus improving its delivering and anticancer activity. Complying with the most modern trends that suggest using nanotechnology, dendrimer nanoparticles were selected as a device to obtain an ETO drug delivery system (DDS) with a more promising Z-potential and an improved DL% in comparison to that previously achieved. In this regard, a fifth generation polyester-based dendrimer (**4**) that was successful in enhancing stability, reducing fast metabolism, and improving several beneficial activity of a natural polyphenol was prepared as previously described [57,58,59,60,61,62].

By using dendrimer **4** for entrapping ETO in its inner cavities, a protective shell around ETO was created. The ETO-loaded nanoparticles that were achieved (namely CPX **5**) showed an improved solubility in solvents where it was already soluble, such as dimethyl sulfoxide (DMSO) (by 1.3 times), but also a highly improved solubility in hydrophilic and biocompatible solvents such as ethanol and water (by 37–390 times). The major water solubility of ETO-loaded nanoparticles might assure, in vivo, a better solubility in gastrointestinal fluids, which represents a crucial property for necessary bioavailability. On the other hand, the preservation of a certain hydrophobic nature will allow a good compatibility with physiologic membranes and, consequently, a higher level of absorbability in the gastrointestinal tract (GIT). CPX showed a spherical morphology, a particle size of about 70 nm, and an optimal Z-potential (−45 mV), matching the values that typically assure low toxicity, high residence time in blood, low polydispersion, and stability in solution [50,51,52]. The drug loading of 37% was determined by UV determinations. The biological activity of CPX **5**, made of the dendrimer scaffold and ETO, was tested on HTLA-230, a high risk MYCN-amplified human neuroblastoma cell line. The unexpected but exciting results revealed that the dendrimer scaffold has a cytotoxic and pro-oxidant effect comparable to that of ETO. Furthermore, it acts in synergy with the complexed ETO, thus increasing its cytotoxic and pro-oxidant action in a time-dependent manner. These events are potentially due to a slow and protracted release of ETO that, entrapped in the dendrimer, is unable to act immediately, leading to a reduced systemic toxicity. The herein prepared ETO reservoir represents a novel biodegradable nanoformulation of ETO with a scaffold-assisted, improved, and sustained cytotoxicity on NB cells.

## 2. Materials and Methods

### 2.1. Chemicals and Instruments

**Scheme 1:**
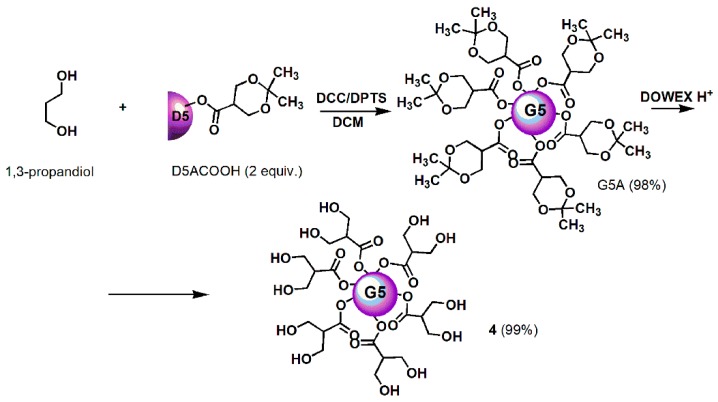
Synthetic procedure for achieving dendrimer **4**. D = Dendron; A = acetonide protected; COOH = free carboxylic group; 5 = mean generations number; DCC = *N*,*N*′-Dicyclohexylcarbodiimide; DPTS = 4-(Dimethyl­amino)pyridinium 4-toluene­sulfonate; DCM = dichloromethane; DOWEX H^+^ = acid resins.

All reagents and solvents were purchased from Merck (formerly Sigma-Aldrich, Darmstadt, Germany). Dendron intermediates were synthetized to achieve dendrimer **4**, i.e., D4BnA, D4BnOH, D5BnA, and D5ACOOH were prepared as previously reported [57,58,59] and their chemical structures are available in Figure S1. Fifth generation polyester-based biodegradable dendrimer **4** was prepared according to the previously reported procedures shown in Scheme 1 (Section 3) [60,61,62]. Its characterization data, including copies of FTIR, ^1^H, and ^13^C NMR spectra, are available in Table S1 and in Figure S2. FTIR and NMR spectra of commercially available ETO are available in Figure S3.

Concerning reagents, no further purification was performed before use, whereas solvents were dried and purified by distillation according to standard procedures. Petroleum ether fraction with a boiling point of 40–60 °C was used in this study. Acetonitrile for UV analysis was of HPLC grade. Triple distilled water (TDW) was used for a saline phosphate buffer (PBS) and for acetonitrile/water medium preparation. PBS and TDW were filtered through 0.22 µm filters (Millipore Sigma Life Science Center, Burlington, MA, USA). Melting points were recorded on a Mettler Toledo MP50 melting points and are uncorrected. FTIR spectra were acquired as films or KBr pellets on a PerkinElmer System 2000 spectrophotometer (PerkinElmer, Inc., Waltham, MA, USA) interfaced to a personal computer (PC), operating under Turbo chrome workstation (version 6.1.1., PerkinElmer, Inc., Waltham, MA, USA). ^1^H and ^13^C NMR spectra were performed on a Bruker Avance DPX 300 spectrometer (Bruker Italia S.r.l., Milan, Italy) at 300 and 75.5 MHz, respectively, and assigned through DEPT-135 and decoupling experiments.

Fully decoupled ^13^C NMR spectra were reported. Chemical shifts were reported in δ (parts per million) units relative to the internal standard tetramethylsilane (δ = 0.00 ppm), and the splitting patterns were described as follows: s (singlet), d (doublet), t (triplet), q (quartet), m (multiplet), and br (broad signal). Centrifugations were performed on an ALC 4236-V1D centrifuge at 3400–3500 rpm. Freeze-drying was performed on an EDWARDS Super Modulyo freeze dryer, ice capacity 8 kg, 8 kg/24 h, refrigeration down to −55 °C with 24-place Drum Manifold. Dynamic light scattering (DLS) and Z-potential determinations were performed on a Zetasizer 3000HS (Malvern Instruments, Malvern, UK) UV-Vis determinations were developed by a double-beam UV-Vis spectrophotometer (Perkin Elmer, Yokohama, Japan), model LAMBDA EZ210 with an automatic wavelength accuracy of 0.1 nm, interconnected to a PC gifted with PESSW software (Version 1.2 and Revision E, Batia InfoTech - Informer Technologies Inc., Los Angeles, CA, USA). Quartz cells of 10 mm path length were employed for containing sample solutions. Scanning electron microscopy (SEM) images were obtained with a Leo Stereoscan 440 instrument (LEO Electron Microscopy Inc., Thornwood, New York, USA). A thin layer chromatography (TLC) system employed aluminium-backed silica gel plates (Merck DC-Alufolien Kieselgel 60 F254, Merck, Washington, DC, USA), and detection of spots was made by UV light. Elemental analyses were determined by an EA1110 elemental analyzer (Fison Instruments, Okehampton, Devon, UK). Organic solutions were dried over anhydrous magnesium sulphate and were evaporated using a rotatory evaporator operating at a reduced pressure of about 10–20 mmHg.

### 2.2. Entrapment Reaction of ETO in Dendrimer **4**

In a test tube flamed under nitrogen gas, ETO (25.0 mg; 0.0425 mmol) was suspended in 1.3 mL of methanol (MeOH) obtaining an ETO suspension with a concentration of 19.2 mg/mL. The solvent was removed at reduced pressure, and the solid residue was dried in vacuum. Separately, a solution of **4** (37.4 mg, 0.0051 mmol) in MeOH (13.0 mL) was prepared with a concentration of 2.9 mg/mL and was added to solid ETO; a total immediate unexpected dissolution was noticed. The solution was stirred in the dark for three days. No precipitate was formed during stirring so filtration was not necessary, and the clear solution was simply evaporated at reduced pressure, obtaining a glassy residue, which was subjected to three cycles of dissolution in acetone and evaporation to remove residual MeOH. The CPX **5** was obtained as a white glassy solid (65.7 mg).

FTIR (KBr, cm^−1^): 3433 (OH of **4** and ETO), 2933, 1735 (C=OO of **4** and ETO), 1540 (ETO), 1506 (ETO), 1485, 1466, 1384 (ETO), 1330 (ETO), 1232 (ETO), 1116 (ETO), 1078 (ETO), 961 (ETO, 932 (ETO), 889, 863, 842 (ETO), 802, 766 (ETO), 701 (ETO), 681 (ETO), 617 (ETO), 598 (ETO). ^1^H NMR (300 MHz, DMSO-*d6*), δ (ppm): 1.01, 1.16, 1.18, 1.23, 1.34 (five s signals, 186H, CH_3_ of generations), 1.70 (m, 2H, CH_2_ propandiol), 3.52 (dd, 128H, CH_2_OH and 2H, CH_2_O propandiol, overlapped signal), 3.61 (signal of ETO), 3.95–4.20 (m, 120H, CH_2_O of four generations and 2H, CH_2_O propandiol, overlapped signal), 4.30 (br s, 64H, OH), 4.94, 5.21, 6.03, 6.18, 6.52, 7.00 and 8.22 (signals of ETO).

### 2.3. Principal Components Analysis (PCA) on FTIR Spectral Data

FTIR analysis of ETO, dendrimer **4**, and CPX **5** were performed by formulating them in KBr pellets. The FTIR spectra were acquired in triplicates for each compound, and the matrix of spectral data of all the spectra acquired were subjected to principal component analysis (PCA) using R statistical software, free downloadable (http://cran.mirror.garr.it/mirrors/CRAN/—Garr Mirror, Milan, Italy).

### 2.4. Quantitative Investigations by UV Spectrophotometric Analysis: ETO Loading (DL%) Determination

The ETO loading determination by spectrophotometric UV analysis was performed according to a method previously described [63], with slight modifications. The medium for developing the analysis was acetonitrile (ACN):TDW 50:50 *v*/*v*. The adopted method had previously been validated by the creators, in terms of concentrations range for linearity, accuracy, selectivity, precision, and repeatability.

#### 2.4.1. Preparation of Calibration Curve

ETO (5 mg) was dissolved in 50 mL ACN, achieving a stock solution of 100 µg/mL. Then, solutions at different concentrations were prepared by transferring aliquots of stock solution into a series of 10 mL volumetric flasks, and volumes were made up with the selected medium. Five different concentrations were prepared, i.e., 5, 10, 20, 30, and 40 µg/mL. ETO estimation was made at 286 nm, and the measurements were obtained against a mixture of ACN/TDW as a blank. Six separate series of solutions of ETO (5–40 µg/mL) were prepared from the stock solution and analyzed, achieving the average absorbance (A_average_) for each ETO concentration ± standard deviation (SD). These data were used to build the standard ETO calibration curve.

The linear regression equation obtained was (1), the regression coefficient (*R*) was 0.999922, and *R*^2^ was 0.9998

*y* = 0.0082*x* − 0.0041 (1)

where *y* is the absorbance (A) measured at λ_max_ = 286 nm and *x* is the ETO concentration (C_ETO_) (µg/mL).

The ETO molar extinction coefficient (ε _ETO_) was computed according to the Lambert–Beer law (2)

A = *ε b M* (2)

where *b* is the optical path length (1 cm) and *M* is the ETO molar concentration and therefore it can be simplified as A = *ε M.*

#### 2.4.2. Estimation of ETO Amount (DL%) Loaded in CPX **5**

By the amount of ETO employed in the complexation reaction and by the amount of CPX **5** achieved, an ETO content of around 38% *w*/*w* was initially estimated. In this regard, in order to test samples with an ETO content value within the linearity range of the calibration curve, 25 mg of CPX **5** were dissolved in ACN (10 mL). An aliquot of 1 mL was diluted to 50 mL with the selected medium (ACN:TDW 50:50 *v*/*v*), obtaining a concentration of 0.050 mg/mL, i.e., 50 µg/mL of CPX, which would mean an amount of ETO around 19 µg/mL. Six aliquots of the so prepared solution were analyzed by UV spectrometer, acquiring the relative absorbance (A) at 286 nm, and the measurements were obtained against a mixture of ACN/TDW as a blank. The molar extinction coefficient of ETO within the complex (ε_ETOC_) was computed according to the simplified version of the Lambert–Beer law (2).

### 2.5. Morphology, Size and Z-potential of CPX **5**

The morphology and size of CPX **5** particles were firstly investigated by scanning electron microscopy (SEM). In addition, the hydrodynamic size (diameter, nm) and Z-potential (mV) of **5** particles were also determined by dynamic light scattering (DLS) analysis for further confirmation of the SEM results.

#### 2.5.1. Scanning Electron Microscopy (SEM)

The sample was fixed on aluminum pin stubs and sputter-coated with a gold layer (30 mA for 1 min) and was examined at an accelerating voltage of 20 kV. The micrographs were recorded digitally using the DISS 5 digital image acquisition system (Point Electronic GmbH, Halle, Germany), and the average size of particles was provided by the instrument.

#### 2.5.2. Dynamic Light Scattering (DLS)

The hydrodynamic size (diameter) of CPX **5**, as well as polydispersion indexes (PdI), were determined in batch mode in a low volume quartz cuvette (pathlength, 10 mm) by a photon correlation spectroscopy (PCS) assembly equipped with a 50 mW He-Ne laser (532 nm) and thermos-regulated at the physiological temperature of 37 °C. The scattering angle was fixed at 90°. Results were the combination of three 10-min runs for a total accumulation correlation function (ACF) time of 30 min. Measurements were performed in PBS as a medium at the maximum concentration of CPX **5** of 3 mg/mL. The hydrodynamic particle size result was volume-weighted and reported as the mean of three measurements ± SD. The PdI value was reported as the mean of 10 measurements ± SD made by the instrument on the sample. The Z-potential was measured at 37 °C in PBS as a medium, and an applied voltage of 100 V was used. The CPX sample was loaded into pre-rinsed folded capillary cells, and twelve measurements were performed.

### 2.6. Evaluation of CPX Solubility

The CPX solubility was determined in DMSO, ethanol (EtOH), and water, reproducing a method previously described for similar polyester-based dendrimer drug formulations and accepted as valid [1]. Briefly, an exactly weighed amount of CPX **5** (9.3 mg) was added with a start aliquot of 250 µL of the selected solvent. Successive aliquots of 250 µL were added (if necessary) up to a maximum of 2 mL (water). The CPX was considered soluble when clear or milky solutions, stable in time, were obtained.

### 2.7. Cell Culture Conditions and Treatments

The MYCN-amplified human stage-IV NB cell line, HTLA-230, was kindly provided by Dr. L. Raffaghello (G. Gaslini Institute, Genoa, Italy). NB cells were treated for 48 and 72 h with 1.25 μM ETO (Calbiochem, Merck KGaA, Darmstadt, Germany) or with the corresponding amount of CPX **5** and dendrimer **4**. The stock solutions of the three compounds were prepared in DMSO, and pilot experiments demonstrated that the final DMSO concentrations did not change any of the cell responses analyzed.

### 2.8. Cell Viability Assay

Cell viability was determined by using the CellTiter 96^®^ AQueous One Solution Cell Proliferation Assay (Promega, Madison, WI, USA). Briefly, cells (15,000 cells/well) were seeded into 96-well plates (Corning Incorporated, Corning, NY, USA) and then treated. Next, the cells were incubated with 20 µL of CellTiter and the absorbance at 570/630 nm was recorded using a microplate reader (EL-808, BIO-TEK Instruments Inc., Winooski, VT, USA).

### 2.9. Detection of Hydrogen Peroxide (H_2_O_2_) Production

The production of H_2_O_2_ was evaluated using 2′-7′-dichlorofluorescein-diacetate (DCFH-DA; Merk Life Science S. r. l. Milan, Italy) according to Negre-Salvayre, et al. (2002) [64]. Briefly, cells seeded (15,000 cells/well) and treated into 96-well plates (Corning Incorporated) were incubated with 5 μM DCFH-DA for 30 min at 37 °C in the dark. At the end, the cells were washed with PBS and incubated with 90% dimethyl sulfoxide for 10 min in the dark with shaking. Fluorescence at 485 nm ex/520 nm em was measured by a fluorimetric plate reader (FLUOstar Optima; BMG Labtech GmbH, Offenburg, Germany). Values were normalized to the protein content and expressed as a percentage of the fluorescence relative to the untreated control.

### 2.10. Statistical Analyses

Data are expressed as means ± SD. Statistical significance of differences was determined by one-way analysis of variances (ANOVA); *p* < 0.05 was considered statistically significant.

## 3. Results and Discussion

### 3.1. Entrapment of ETO in Dendrimer **4**

The reduced bioavailability, the induction of several toxic effects, and the reduced selectivity towards cancer cells limit the efficacy of chemotherapeutic drugs. For this reason, there is a strong incentive for the development and improvement of nanoparticles containing drugs. Regarding ETO, appealing inorganic nanohybrid constructs [50] have been successfully developed but, according to the parameters typically associated with a good drug delivery system, a number of improvements have been considered feasible. The Z-potential of +20.5 mV suggests a certain instability in solution, with a tendency towards aggregations and some residual toxicity due to the positive value that could be canceled with higher and negative Z-potentials [51,52,53]. In addition, an improved DL could be achieved by exploiting dendrimers, which are nano-carriers known to be endowed with a greater loading capacity. Dendrimers are highly branched and symmetric nanosized macromolecules and are characterized by a monodisperse structure miming the tree’s ramification, with both internal cavities for guest molecule entrapment and many peripheral functional groups, which offer the possibility of a further functionalization by covalent bond [1]. They differ from traditional polymers because they have an uncommon low intrinsic viscosity that allows their easy transport in the blood [2,3], and they are characterized by a very low PdI of <1.1 or are even monodispersed [4]. When used as delivery devices for drugs, they can control their molecular weight, hydrophilicity, solubility, bioavailability, and pharmacokinetic behavior [4,5,6]. Thanks to their ability to establish strong interactions with several drugs, dendrimers are endowed with easily realizable high drug loading, thus limiting drug systemic toxicity by minimizing the initial massive drug release when parenterally administered [4,5,6]. Consequently, dendrimers, mainly biodegradable polyester-based ones, are considered to be excellent carriers for drug delivery. In this regard, a fifth generation polyester-based dendrimer (namely **4**), with a high molecular weight, was selected as a carrier to entrap ETO and achieve an ETO reservoir with improved physicochemical and therapeutic properties. The choice was justified by virtue of the dendrimers’ unique properties. As reported [65], the internal architecture of a dendrimer is usually hydrophobic, thanks to hydrophobic interactions and hydrogen bond formations, and so it would be suitable for ETO, which is a hydrophobic compound. High generation dendrimers, such as **4**, are endowed with more space to host hydrophobic drugs and are more promising for a high drug loading [66], and the big constructs with large surfaces and high molecular weight obtainable with such carriers are typically retained in the circulation for longer periods [67].

They behave as ‘excipients’ or permeability enhancers, they alter the barrier function of the intestinal epithelium, and enhance the permeability of the co-administered drug [67]. Non-covalent drug complexation, rather than covalent surface linkage, is the preferred technique for enhancing the solubility and bioavailability of several drugs [66] to provide them with a protective shell that is able to limit inactivating processes. The uncharged polyester-based hydrolysable architecture of **4**, known to be biodegradable [4,55,56], allows for the reduction of the risk of inducing cell death by irreversible cell membrane damage, which is commonly associated with the use of PAMAM and *b*-PEI structured matrices.

#### 3.1.1. Chemistry

Dendrimer **4**, selected as host carrier to complex ETO, was prepared as previously described, according to Scheme 1 [60,61,62].

**Scheme 2:**
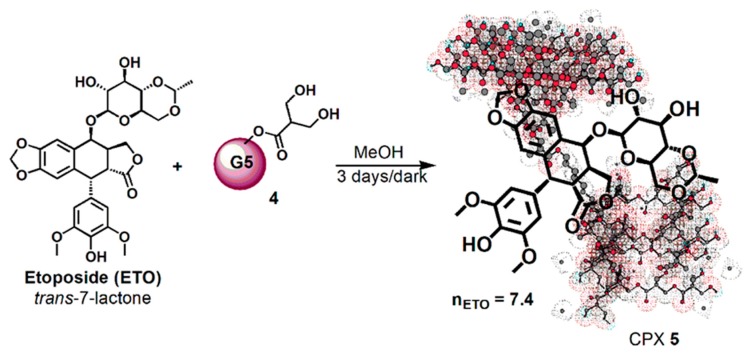
Synthetic procedure for entrapping ETO in dendrimer **4**.

Then, dendrimer **4** dissolved in MeOH was vigorously stirred for 72 h at room temperature in the dark with ETO (Scheme 2), achieving the complex CPX **5**, which has the intuitive structure shown in Scheme 2. The tridimensional structure of the host dendrimer (**4**) was obtained with the three-dimensional Chem Draw software (PerkinElmer ChemDraw^®^, Milan, Italy).

Considering that ETO is completely not soluble in MeOH, the remarkable solubilizing action of dendrimer **4** was evident from the initial phase of the reaction. By the addition of a methanol solution of **4** to solid ETO, it immediately dissolved, improving its original reported max solubility (1 mg/mL) two fold. No precipitate was formed during the three days under stirring in the dark, and finally CPX **5** was recovered by the reaction solution through the simple removal of MeOH at reduced pressure and acetone washings. The CPX **5** was obtained in the form of a white glassy solid (65.7 mg) and was stored on P_2_O_5_ in a dryer at r.t. Since ETO was totally complexed and entrapped in dendrimer **4**, from its initial weight of 25.0 mg and that of CPX **5** (65.7 mg), a drug loading (DL%) of 38% was preliminarily estimated.

CPX **5** was firstly qualitatively characterized by FTIR spectroscopy, whose data were also processed by principal component analysis (PCA), and NMR analysis. UV-Vis spectrophotometric analysis was selected for the quantitative determination of the ETO amount entrapped in the CPX **5** and to compute the drug loading (DL%) and the entrapment efficiency (EE%). Finally, CPX particle morphology, size, polydispersion index (DpI), and Z-potential were determined by SEM and DLS analysis.

#### 3.1.2. FTIR Characterization

**Figure 4:**
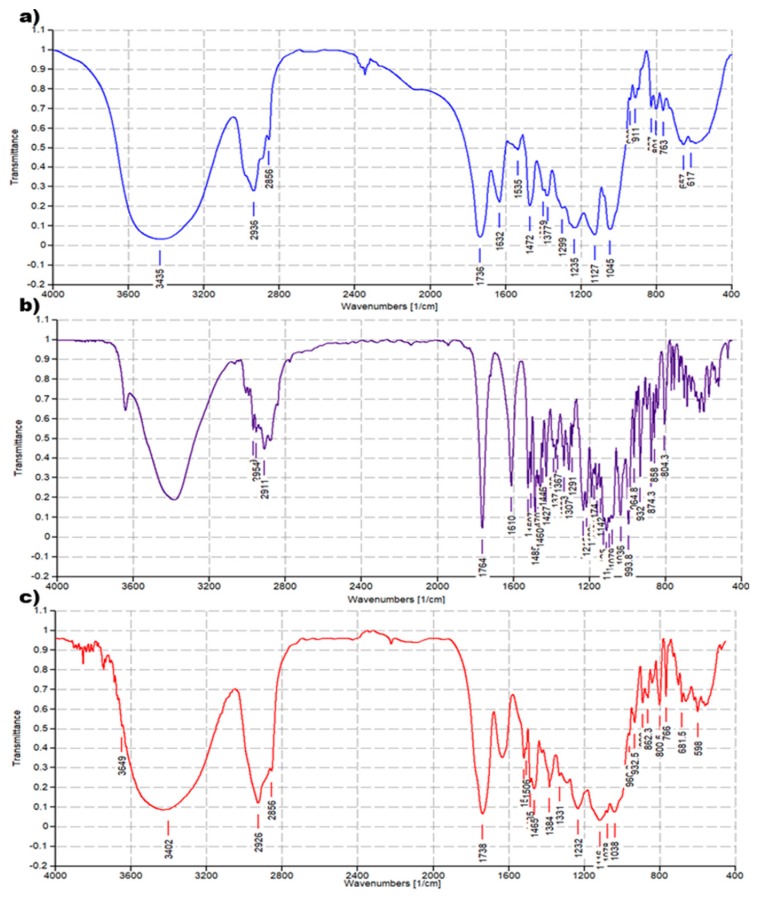
Comparison between FTIR spectrum of dendrimer **4** (**a**), ETO (**b**), and CPX **5** (**c**).

The FTIR analysis of CPX **5** was performed by preparing KBr pellets containing **5** and acquiring the FTIR spectra in triplicate. The absorption peaks of CPX **5** appeared as shown in Figure 4c. Another copy of the FTIR spectra of **5** is available in the SM (Figure S4a). For comparison purposes, FTIR spectra of **4** (Figure 4a) and ETO (Figure 4b) were also acquired in the same condition, and other copies of the obtained spectra are available in the SM (Figure S2a and Figure S3a). FTIR analysis was significantly diagnostic to confirm the successful complexation of ETO with **4**. As shown in Figure 4 and in Figure S5, in the FTIR spectrum of CPX **5**, many bands belonging to ETO and absent in the spectrum of **4** are well evident, thus confirming the presence of ETO complexed to the hosting dendrimer **4**. The peak lists obtained from the spectra in Figure 4 and in Figure S5 have been reported below, with the significant peaks indicating the success of the entrapment reaction underlined.

**ETO**. FTIR (KBr, cm^−1^): 1764, 1610, 1538, 1507, 1485, 1470, 1460, 1446, 1427, 1389, 1377, 1367, 1333, 1307, 1291, 1231, 1215, 1188, 1174, 1161, 1142, 1125, 1116, 1084, 1079, 1036, 994, 965, 949, 932, 913, 896, 874, 858, 839, 768, 753, 728, 700, 685, 664, 619, 598, 570, 519.

**Dendrimer 4**. FTIR (KBr, cm^−1^): 1737, 1631, 1536, 1472, 1376, 1236, 1128, 1045, 912, 827, 801, 763, 657.

**CPX 5**. FTIR (KBr, cm^−1^): 1735, 1540, 1506, 1485, 1466, 1384, 1330, 1232, 1116, 1078, 961, 932, 889, 863, 842, 802, 766, 701, 681, 617, 598.

#### 3.1.3. NMR Characterization

**Figure 5:**
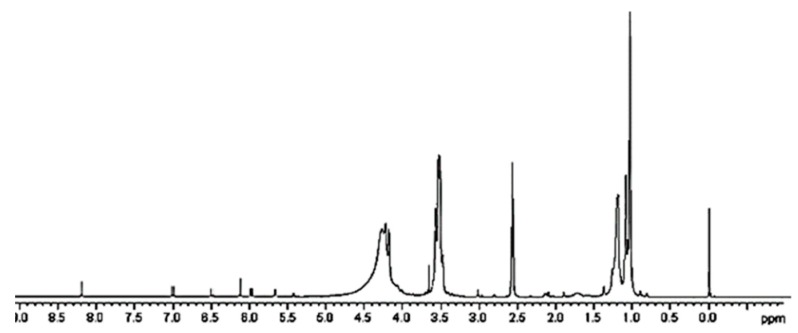
^1^H NMR of CPX **5** (DMSO-d6, 300 MHz).

A further confirmation of the successful entrapment of ETO in **4** was provided by ^1^H NMR spectroscopy. While the peaks belonging to ETO proton atoms are present in the spectrum region from 1.24 to 8.22 ppm (Figure S6a), dendrimer **4** signals are not existing after 4.5 ppm (Figure S6b). Looking at the spectrum of complex **5** (Figure 5 and Figure S6c), in the region 1–4.5 ppm, the rather wide and intense peaks of the dendrimer polymer **4** are detectable and completely cover the possible ETO signals. Only one peak around 3 ppm, and not sufficient to attest ETO presence, is detectable. Differently, in the spectrum region above 4.5 ppm, even if of very low intensity, seven small signals perfectly coinciding with ETO signals and absent in the spectrum of **4** are clearly visible.

Usually, ^1^H NMR analysis is also useful for obtaining quantitative evaluations and determining DL%. In this case, in order to quantify the ETO content in CPX **5**, integrals related to very different numbers of proton atoms should have been compared, committing substantial errors. UV spectrophotometric determination was used in place of NMR to assess the ETO content in CPX **5**.

#### 3.1.4. Principal Components Analysis (PCA) on FTIR Spectral Data

In order to get a more reliable information concerning CPX **5** chemical composition from FTIR spectral data, it was of great help to resort to a specific analytical technique that was able to work on very complex data sets, known as principal component analysis (PCA). This technique can reduce multi-dimensional data to a small number of new variables—principal components (PCs) [68]. A more detailed description of the PCA chemometric tool is available in the Supplementary Materials Section S5. In this work, the data of the nine spectra obtained by the FTIR analysis made in triplicate on the three compounds under study (**4**, **5**, and ETO), were arranged into a matrix of variables that was subjected to PCA. Ninety-nine percent of the explained variance was provided by PC1 and PC2, and the results are reported as bi-plot and score plot in Figure S7. The desired information was clearly observable on PC2. In this regard, both in bi-plot and score plot, by observing the relative positions of **4**, **5**, and ETO on PC2, it appears that **5** is located in the middle between **4** and ETO and slightly closer to **4** rather than to ETO. Such disposition suggests that the chemical composition of **5** is a mixture of ETO and **4** in a ratio slightly inferior to 50% and confirms the presence of ETO inside the complex.

#### 3.1.5. Quantitative Investigations by UV Spectrophotometric Analysis: ETO Loading (DL%) Determination

For a correct drug content estimation, sensitive, simple, and cost effective methods are needed, including UV, visible, fluorescence, and high performance liquid chromatography. For the estimation of ETO, the most reported methods are based on liquid chromatographic technique utilizing UV [69,70], fluorescence [71], and electrochemical [72] detectors or the HPLC method utilizing gradient technique (USP, 2003). These reported methods are tedious, expensive, complicated, and involve lengthy extraction procedures [73,74,75]. Simple UV and spectrofluorimetric methods have been found to be very suitable for the estimation of drug content, entrapment efficiency, and in vitro release studies. Therefore, a previously reported UV method [63], slightly modified, has been adopted in this work to estimate ETO content in CPX **5**.

##### Calibration Curve

Similarly to the value reported for the UV spectrum of ETO acquired in ACN [63], i.e., λ = 285 nm, the UV spectra of ETO acquired in this study in ACN/TDW 50:50 showed a well-defined maximum at λ = 286 nm (Figure S8a), and this wavelength was chosen as the analytical wavelength to build up the standard ETO calibration curve and then to quantify the amount of ETO in CPX **5**. The UV spectra of ETO in the selected medium at the different concentrations are shown in Figure S8a. Six separate series of standard solutions of ETO at concentrations in the range 5–40 µg/mL (identified as the linearity range) were prepared and analyzed by reading absorbance at λ = 286 nm. The average absorbance (A_average_) values ± SD measured for each concentration of ETO (C_ETO_) (*n* = 6) have been reported in Table S2.

A_average_ and C_ETO_ (µg/mL) data in Table S2 were used to build up the standard ETO calibration curve shown in Figure S8b whose Equation (1) has been reported in the Material and Methods (Section 2.4.1) and whose very high coefficient of correlation (*R*) assured a condition of linearity. The standard error of prediction was 0.2064, and the predicted ETO concentrations (C_ETOp_) were calculated for each sample and reported in Table S2 together with the residuals. The real concentrations versus the predicted ones are reported in a graph (Figure S8c), and the linear regression equation correlating the two data sets is observable in Figure S8c. The coefficient of correlation *R* was 0.99993, and *R*^2^ was 0.99998.

A_average_ measured and ETO µM concentrations (last column in Table S2) were used to calculate the molar extinction coefficient of ETO (ε_ETO_), according to the simplified version of the Lambert–Beer law (2), usable when the optical length path is 1 cm.

Briefly, A_average_ values versus C_ETO_ concentrations (µM) were plotted on a graph (Figure S8d), and it was verified that the intercept was not significantly different from zero. Consequently, the linear regression equation with intercept zero obtained was (3), the regression coefficient (*R*) was 0.99992, and *R*^2^ was 0.9994.

*y* = 0.0047*x* (3)

where *y* is the absorbance (A) measured at λ = 286 nm and x is the C_ETO_ (µM).

In a graph of A versus C, the slope is the molar extinction coefficient (ε) of the compound under study. In this regard, the molar extinction coefficient of ETO (ε_ETO_) was 0.0047 µM^−1^ L cm^−1^, i.e., 4700 M^−1^ L cm^−1^.

##### Estimation of ETO Amount in CPX **5** (DL%) and of Entrapment Efficiency (EE%)

Six aliquots of a solution of CPX **5**, prepared at a concentration of 50 µg/mL, were analyzed by UV spectrometer, acquiring the absorbance at λ = 286 nm. By using Equation (1), the correspondent concentrations of ETO were determined, and by using the simplified form of the Lambert–Beer law 2 the correspondent molar extinction coefficients of ETO within the complex (ε_ETOC_) were computed. The measured absorbance (A), the relative ETO concentrations (C_ETO_), and the molar extinction coefficients of ETO within the complex (ε_ETOC_) for each aliquot have been reported in Table 1.

The average absorbance (A_average_) resulted to be 0.149 ± 0.0021, and the average ETO concentration (C^ETO^_average_) in the sample of CPX **5** analyzed, resulted to be 18.7 ± 0.24 µg/mL. The drug loading (DL%) was calculate by:

$$\mathrm{DL}\%\;=\;\frac{\mathrm{Weight}\;\mathrm{of}\;\mathrm{etoposide}\;\mathrm{in}\;\mathrm{CPX}\;}{\mathrm{Weight}\;\mathrm{of}\;\mathrm{CPX}}\;\times 100$$

It resulted to be 37.4% *w*/*w* and was therefore both very satisfying and remarkably improved in respect of ETO nanocomposites prepared by Qin et al. [50].

The molar extinction coefficient of ETO within the complex (ε_ETOC_) at the obtained concentration of 18.7 µg/mL, calculated by the Lambe–Beer law, was 4690 M^−1^ L cm^−1^. The molar extinction coefficient of free ETO (ε_ETO_) provided by Equation (3) was 4700 M^−1^ L cm^−1^, thus demonstrating that there has been no significant variation in the drug extinction coefficient with its complexation and thus validating the UV proposed method. For further confirmation, ETO concentration (µg/mL) was also calculated by using the molar extinction coefficient of the not complexed ETO (0.0047 µM^−1^ L cm^−1^) and the simplified form of the Lambert–Beer law 2. The result was C_ETO_ = 31.7 µM, i.e., 0.0317 µmol/mL and 18.66 µg/mL, which is within the limit values of the ETO concentration obtained through the calibration curve (18.46–18.94).

Once the content of ETO for a given weight of CPX **5** was known, the ETO moles (moles_ETO_) loaded per dendrimer **4** mole (mole**_4_**) were calculated, and from this data the molecular weight (MW) of **5** was computed (Table 2). In particular, MW was calculated according to a procedure previously reported in the literature and accepted as valid [1,5,6] without resorting to widely used but very expansive methods such as Matrix-Assisted Laser Desorption/Ionization-Time Of Flight (MALDI-TOF) mass spectrometry (MS). In addition, the ETO entrapment efficiency (EE%), which is defined by the concentration of the incorporated material detected in the formulation over the initial concentration used to make the formulation, was also calculate by:

$$\mathrm{EE}\%\;=\;\frac{\mathrm{Weight}\;\mathrm{of}\;\mathrm{etoposide}\;\mathrm{in}\;\mathrm{CPX}\;}{\mathrm{Weight}\;\mathrm{of}\;\mathrm{ETO}\;\mathrm{fed}\;\mathrm{initially}}\;\times 100$$

The result was found to be 89.3% and has been reported in Table 2. Concerning ETO-loaded nanoparticles prepared in the last decade, only very high dimensioned pegylated liposomal ETO nanoparticles of 491 nm, characterized by a limited DL% of 10%, proved to have a very high entrapment efficiency of 99% [76]. In other cases, the ETO EE% was either very low (4–14%) [77] or, though high (75–84%) [78], were not as high as the EE% achieved in this study by using dendrimer **4**. Only recently, Thiyagarajan et al. [79], have prepared a series of polymeric nanoparticles containing ETO for targeting cancer cells that had a high DL% (62–90%) and in some cases very high EE% (49–94%).

#### 3.1.6. Morphology, Size, and Z-potential of CPX **5**

**Figure 6:**
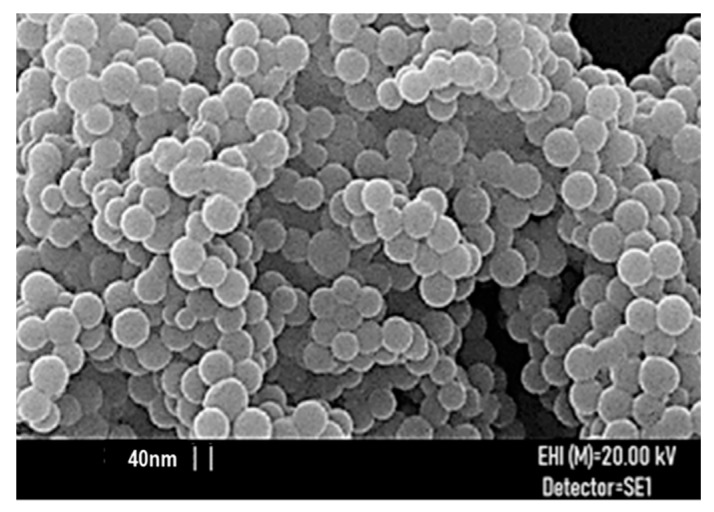
SEM image of CPX **5** particles.

The SEM image of CPX **5** particles is shown in Figure 6, while the average particle size, provided by the instrument, is reported in Table 3.

For a good functioning of a nano delivery system, the size and morphology of nanoparticles play a key role. These features have a great influence on a drug delivery system’s distribution, toxicity, targeting ability, and drug release profile [80]. For biomedical applications, the preferential size has to be less than 200 nm, with an optimal of 100–200 nm for assuring an efficient cellular up-take [81]. In this regard, in a reported study [82], drug delivery systems with 100 nm nanoparticles exhibited a 2.5-fold greater uptake compared to 1 µm diameter particles and a 6-fold greater uptake than 10 µm particles. Particles of 200 nm or larger tend to activate the lymphatic system and are removed from circulation quicker [81]. Particle size of about 100 nm encompass larger surface area to volume ratio and allow a more effective and fast drug release because more of the drug is closer to the surface of the particle [83]. In addition, particles at a dimension of 100 nm could also pass through the blood-brain barrier (BBB) and deliver sufficient amounts of drug, avoiding immediate clearance by the lymphatic system [80].

CPX **5** particles in SEM images show a spherical morphology, which suggests a very large surface area that typically increases the drug delivery system’s (DDS’s) systemic retention time and positively affects their bio-efficiency [67,80]. The mean particle size of **5** was 76 nm, i.e., slightly inferior to 100 nm, which is reported as the ideal size value for nanoparticle drug formulations suitable for biomedical applications [80].

Concerning nanomaterial sized at values <100 nm, McMillian et al. [84] reported that, thanks to their physical properties, they possess a unique biologic potential for biomedical applications. Such particles, and therefore the ones of CPX **5**, represent attractive systems for the treatment of cancer and heart and lung, blood, and inflammatory disorders, and infectious diseases, including central nervous system disorders.

For further confirmation of the SEM results, the particle hydrodynamic size (diameter, nm) of CPX **5** was measured using DLS analysis, and the result matched the SEM ones with a very small error of 7 nm. It was reported as the intensity-weighted average (Z-AVE) [N (degree of freedom) = 3] in Table 4, and size distribution has been shown in Figure S9. The PdI value (*n* = 10) was 0.5 and was therefore in the range of PdI recently reported for promising ETO nanoparticles formulations [79]. In addition, by DLS technique, the Z-potential (mV) of CPX **5** particles was also measured, for the purpose of having an idea of physical stability of **5** in aqueous solution, for investigating the possible tendency of the particles to form aggregates, and for finding its possible toxicity. The result has been reported in Table 3.

Z-potential is the potential difference between the dispersion medium and the stationary layer of fluid attached to particles. It is a measure of the electrical charge of particles that are suspended in liquid and is also crucial for nanoparticles-cell interactions. Low Z-potential favors the particles binding to low serum proteins and a longer circulation in blood. Values <5 mV may lead to agglomeration of particles conferring physical instability in solution. On the contrary, Z-potential >30 mV, either positive or negative, leads to monodispersity and good physical stability in solution [45,46] Finally, high positive Z-potential values are correlated to high cytotoxicity while negative values allow high cell viability [47]. In this regards, the CPX **5** nanoparticles that proved to possess a Z-potential of −45 mV ± SD can be rationally considered stable in water solution, with no tendency to form aggregates and are endowed with low cytotoxicity.

#### 3.1.7. Evaluation of CPX **5** Solubility

According to a reported method used to evaluate the solubility of two ellagic acid-loaded polyester-based dendrimers [1], the CPX **5** solubility was determined in DMSO and in the commonly biocompatible hydrophilic solvents used for biomedical applications, i.e., EtOH and water. DMSO dissolves very well both free ETO and CPX **5**, but it is known to be hazardous for humans and not advisable for clinical applications. CPX **5** solubility in DMSO was, however, assessed to have an ideal maximal concentration achievable in biological studies on cell models, usually performed in DMSO, as in this study. Concerning biocompatible solvents, free ETO is poorly soluble in EtOH and practically non-soluble in water. By its entrapment inside the dendrimer architecture of **4**, ETO solubility was improved in all the solvents considered. CPX **5** resulted to be more soluble than ETO by 48 times in EtOH and by 162–392 times in water (Table 4).

CPX **5** gave clear and stable solutions in DMSO and EtOH, while a fine suspension with pH = 7.4, stable along time, was achieved in water. Water fine suspension, obtained at a concentration of 4.7 mg/mL, became clear with the addition of 150 μL of EtOH. Biocompatible ethanol, water/ethanol solutions, and water suspensions of CPX **5** can be considered suitable for oral administration of ETO.

### 3.2. Biological Activity of CPX **5** on Human NB Cells

#### 3.2.1. CPX **5** Revealed a Synergistic Cytotoxic Effect Exerted by Dendrimer **4** per se and by the Complexed ETO Slowly Released over Time

**Figure 7:**
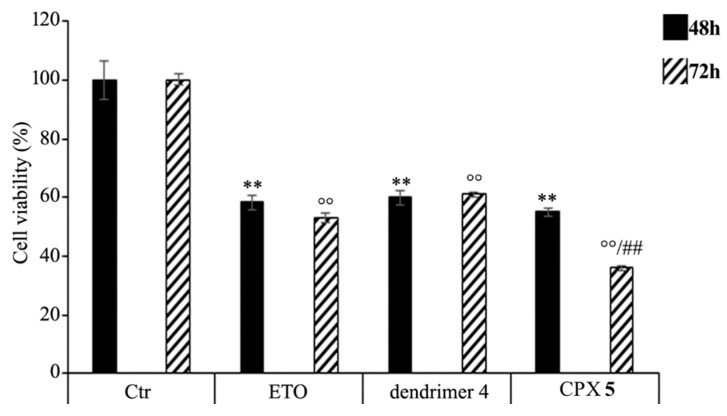
Cell viability was evaluated by CellTiter 96^®^ AQueous One Solution Cell Proliferation Assay in NB cells exposed to ETO (1.25 μM), CPX **5** (in a dose able of providing 1.25 μM ETO, and dendrimer 4 (at the same dose of CPX **5**) for 48 and 72 h. Histograms summarize quantitative data of the means ± S.E.M. of three independent experiments. ** *p* < 0.01 vs. Ctr cells (48 h); °° *p* < 0.01 vs. Ctr cells (72 h); ## *p* < 0.01 vs. ETO-treated cells (72 h).

HTLA-230 NB cells were treated with 1.25 μM ETO, CPX **5** (in a dose capable of providing 1.25 μM ETO), and dendrimer **4** (at the same dose of that contained in the administered CPX **5**) for 48 and 72 h, and the effect of all treatments on NB cell viability was investigated. After 48 h of exposure to ETO, dendrimer **4**, or CPX **5**, the effects were comparable, since all single treatments reduced NB cell viability by 40–45%. Interestingly, comparing the effects induced by the 72 h treatment, CPX **5** was able to significantly reduce cell viability by 65% in respect to untreated cells and by 30% in respect to ETO-treated cells (Figure 7).

These results demonstrate that the ETO entrapment in dendrimer **4**, although with a delayed effect, increases the cytotoxicity of the drug, and this effect is probably the result of two mechanisms that act synergistically. On one hand, CPX **5** guarantees a slow and protracted release of ETO over time, whereas, on the other hand, it adds a greater cytotoxic effect of the host dendrimer (**4**) to that of the delivered ETO. A similar result has been observed in another study employing ETO-loaded poly (lactide-co-glycolide) nanoparticles in which a sustained release of the drug was maintained until 72 h [88]. In addition, it has been reported that pegylated liposomal nanoparticles, containing a non-clinically useful ETO concentration, had a greater cytotoxic effect than that of the free drug [89]. However, in our experimental model, CPX **5** would appear to be a more efficacious delivery system because it significantly increases the cytotoxic effect of an ETO dose comparable to that commonly used to treat NB patients [90].

#### 3.2.2. CPX **5** Potentiates the Cytotoxic Action of ETO by Increasing Reactive Oxygen Species (ROS) Production

**Figure 8:**
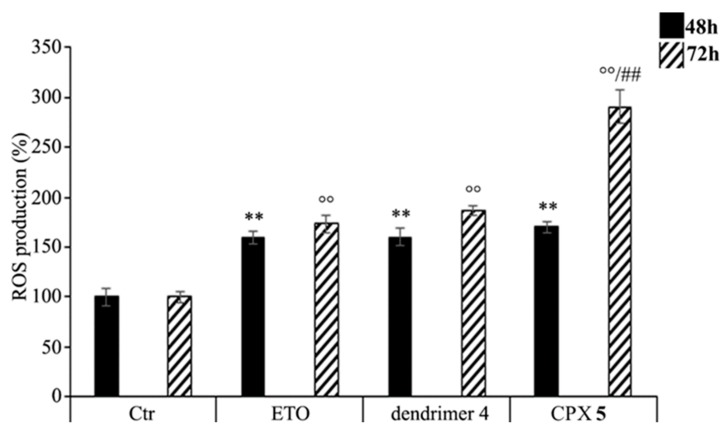
Reactive oxygen species (ROS) generation was analyzed in neuroblastoma (NB) cells exposed to ETO (1.25 μM), CPX **5** (in a dose able of providing 1.25 μM ETO), and dendrimer **4** (at the same dose of CPX **5**) for 48 and 72 h. Histograms summarize quantitative data of the means ± SEM. of three independent experiments. ** *p* < 0.01 vs. Ctr cells (48 h); °° *p* < 0.01 vs. Ctr cells (72 h); ## *p* < 0.01 vs. ETO-treated cells (72 h).

As shown in Figure 8, CPX **5** treatment increased the production of ROS in a time-dependent manner. In particular, compared to control cells, ROS levels were increased by 70% and 190% after 48 and 72 h, respectively, while the treatment with free ETO or dendrimer **4** stimulated ROS production by 60% and 80% after 48 and 72 h (Figure 8). To our knowledge, ETO is well known to exert its cytotoxic action by increasing ROS production [91,92], while a pro-oxidant effect of nanoparticles was reported only for polyamidoamine dendrimers (PAMAMs) [93]. Our present data shows that dendrimer **4**, which was able per se to increase ROS production, when included in the formation of CPX **5** markedly enhanced the pro-oxidant action of ETO, thus creating conditions of oxidative stress capable of triggering cell death (Figure 7).

Though the here presented results are already considerable, for the future, further improvement could be achieved by a partial esterification of peripheral hydroxyls of **4** with targeting molecules such as folic acid that is recognized by cancer cells for a more selective action and a reduced toxicity for healthy cells.

## 4. Conclusions

As in the case of many chemotherapeutic drugs, also for ETO, the efficacy is often limited by the reduced bioavailability, the induction of several toxic effects that forces to limit dosage, the metabolism or environmental-induced inactivation, and the reduced selectivity towards cancer cells.

An appealing strategy, currently being studied and which is giving promising results, is the administration of chemotherapeutic drugs entrapped in nanoparticles, including the well-known dendrimers endowed with nonpareil properties.

In this context, ETO has been successfully combined with a lab-made biodegradable dendrimer nanoparticles (**4**) that, being uncharged differently from the well-known PAMAMs, are respectful to cell membranes and do not provoke irreversible cell damage. It is important to note that the structure and the chemical composition of the ETO-loaded dendrimer achieved (CPX **5**) has been qualitatively and quantitatively investigated by FTIR, NMR, and UV spectroscopy, and a more in depth interpretation of FTIR spectral data by performing PCA has been obtained and provided. In addition, by SEM and DLS analysis, CPX **5** showed a spherical morphology, a particle size of about 70 nm, and a Z-potential of −45 mV, matching the values that typically assure low toxicity, low poly dispersion index, high residence time in blood, and stability in solution. The drug loading was 37%, the entrapment efficiency was 89%, and the solubility of ETO in biocompatible solvents was amazingly improved (390 times in H_2_O). Experiments performed to assess CPX **5** bioactivity on NB cells in comparison with ETO and dendrimer **4** alone demonstrated unexpectedly that the hosting dendrimer **4** per se is able to exert a ROS-mediated cytotoxicity comparable to that of free ETO. Interestingly, in CPX **5**, the bioactive scaffold of dendrimer **4** has been found to act in synergy with the complexed ETO that is released in a protracted manner, determining a significant improvement of the cytotoxic activity of free ETO. In addition, having a high drug loading and providing the hosted ETO with a protective shell, CPX **5** can guarantee an improvement of the ROS-mediated cytotoxic action of ETO for a long time. Consequently, although preliminary, our data suggests that CPX **5**, endowed with a considerably improved water solubility and a significant scaffold-assisted anti-cancer activity, can become a promising formulation for the protracted release of ETO in order to sensitize NB cells. In conclusion, collectively, these results suggest that dendrimer **4**, having per se a ROS-mediated cytotoxicity, could be considered an efficient chemiosensitizer that, inducing oxidative stress, weakens cancer cells facilitating the trigger of drug-induced oxidative death. The already very interesting and satisfying results obtained by combining ETO with the not functionalized dendrimer **4** could be further improved by the partially esterification of the peripheral hydroxyls of **4** with targeting molecules such as folic acid that is recognized by cancer cells for a more selective action and reduced toxicity for healthy cells.

## Figures and Tables

**Table 1:** Values of A, C_ETO_, and ε_ETOC_ obtained for the six aliquots of a 50 µg/mL sample of CPX **5**.

A	C_ETO_ (µg/mL)	ε_ETOC_ (M^−^^1^ L cm^−1^)
0.149	18.7	4690
0.152	19.0	4709
0.146	18.3	4696
0.149	18.7	4690
0.148	18.6	4684
0.151	18.9	4703

**Table 2:** Estimated ETO amount in a 50 µg/mL sample of CPX **5** expressed in µg, as DL% *w*/*w*, as EE% w/w, and as ETO moles (moles_ETO_) loaded per dendrimer **4** mole (mole**_4_**), and computed molecular weight (MW) of CPX **5**.

Dendrimer 4 (µg, µmol)	ETO Content (µg, µmol)	DL% (*w*/*w*)	EE% (*w*/*w*)	ETO Content (moles_ETO_/mole_4_)	Molecular Weight (MW) of CPX 5
31.3, 0.0043	18.7, 0.032	37.4	89.3	7.4	11,631

**Table 3:** CPX **5** particle hydrodynamic size, PdI, and Z-potential at 37 °C by dynamic light scattering (DLS) analysis and average particles size by SEM analysis.

Z-AVE Size (nm) ^1^	PdI ^2^	Average Particles Size (nm) by SEM Analysis	Z-potential (mV) ^3^
69.2 ± 1.08	0.536 ± 0.002	76.25 ± 17.3	−45 ± 0.34

^1^ Z-AVE size determinations = 3. ^2^ PdI determination = 10. ^3^ Z-potential determinations = 12.

**Table 4:** Comparison between solubility of free ETO and ETO-loaded CPX **5**.

Solvent	ETO	CPX 5	Improvement (times)
DMSO	max 58.9 mg/mL [85]	78.8 mg/mL	1.3
EtOH	0.78 mg/mL [86]	>37.2 mg/mL	>47.7
H_2_O	0.012–0.029 mg/mL [86,87]	4.7 mg/mL	162.1–391.7

## Supplementary Materials

The following are available online at https://www.mdpi.com/2076-3921/9/1/50/s1, Figure S1: Structure of dendron intermediates achieved to synthetize **4**: D4BnA, D4BnOH, D5BnA, and D5ACOOH, Table S1: Molecular weight (MW) and significant physicochemical data of dendrimer **4**, Figure S2: Copies of FTIR (a), ^1^H NMR spectrum (b) and ^13^C NMR and DEPT 135 spectra (c) of dendrimer **4**, Figure S3: Copies of FTIR (a), ^1^H NMR spectrum (b) and ^13^C NMR spectrum (c) of ETO, Figure S4: Copies of FTIR (a) and NMR (b) spectra of CPX **5**, Figure S5: FTIR spectra of ETO (a), dendrimer **4** (b), and CPX **5** (c) with, in evidence, peaks of **4** (1), peaks of ETO (2), and peaks of **5** (3), Figure S6: ^1^H NMR spectra of ETO (a), dendrimer **4** (b), and CPX **5** (c), Figure S7: Bi-plot and score plot of Components PC1 and PC2, Table S2: Data of the calibration curve: A_average_ and ETO standards µg/mL concentrations. ETO predicted concentrations, residuals, and ETO µM concentrations, Figure S8: UV spectra of ETO dissolved in ACN/TDW **5**0:50 at the different concentrations used to build up the standard ETO calibration curve (a), standard ETO calibration curve (b), real ETO concentrations versus predicted ones (c), Absorbance (A) at λ = 286 nm versus standards ETO concentrations (µM) (d), Figure S9: Dynamic light scattering analysis of CPX **5**.
