# Supplementary material for: Polyester-Based Dendrimer Nanoparticles Combined with Etoposide Have an Improved Cytotoxic and Pro-Oxidant Effect on Human Neuroblastoma Cells
Source: Antioxidants (Basel). 2020 Jan 6;9(1):50. doi: 10.3390/antiox9010050 (PMC7022520; doi:10.3390/antiox9010050)
Supplement: Supplementary file 1 [file antioxidants-09-00050-s001.pdf]

antioxidants

Supplementary Material

Polyester-based dendrimer nanoparticles combined with Etoposide have an improved cytotoxic and pro-oxidant effect on human neuroblastoma cells

Silvana Alfei,[1,*,§] Barbara Marengo,[2,§] and Cinzia Domenicotti[2,*]

[1]Department of Pharmacy, University of Genoa, Viale Cembrano, 4 I-16148 Genoa, Italy
[2]Department of Experimental Medicine - DIMES, Via Alberti L.B. 2 I- 16132 Genoa, Italy

[§] Authors have equally contributed

*Corresponding Authors: cinzia.domenicotti@unige.it, alfei@difar.unige.it

Table of Contents

Figure S1: Structure of dendron intermediates synthetized to prepare **4**: D4BnA, D4BnOH, D5BnA and D5ACOOH

Section S1: Characterization of dendrimer **4**

Table S1: Molecular Weight (MW) and significant physicochemical data of dendrimer ***4***

Copies of FTIR and NMR spectra of dendrimer ***4***

Figure S2: FTIR of dendrimer ***4*** *(a), ^{1}H NMR of dendrimer* ***4*** *(b), ^{13}C NMR and DEPT 135 of dendrimer* ***4*** *(c)*

Section S2: FTIR and NMR spectra of etoposide (ETO)

Figure S3: FTIR of ETO (a), ^{1}H NMR of ETO (b), ^{13}C NMR of ETO (c)

Section S3: FTIR and NMR spectra of CPX **5**

Figure S4: FTIR of CPX ***5*** *(a), ^{1}H NMR of CPX* ***5*** *(b)*

Section S4: Comparison between FTIR and ^{1}H NMR spectra of ETO, dendrimer **4** and CPX **5**

Figure S5: FTIR spectra of ETO (a), dendrimer ***4*** *(b) and CPX* ***5*** *(c) with in evidence peaks of* ***4*** *(1), peaks of ETO (2) and of* ***5*** *(3)*

Figure S6: ^{1}H-NMR spectra of ETO (a), dendrimer ***4*** *(b) and CPX* ***5*** *(c)*

Section S5: Principal Components Analysis Results

Figure S7: Bi-plot and score plot on Components PC1 and PC2

Section S6: UV Spectrophotometric Analysis results

Table S2: Data of the calibration curve: $A_{average}$ and ETO standards μg/mL concentrations. ETO predicted concentrations, residuals and ETO μM concentrations

Figure S8: UV spectra of ETO dissolved in ACN/TDW 50:50 at the different concentrations used to build up the standard ETO calibration curve (a), standard ETO calibration curve (b), real ETO concentrations versus predicted ones (c), Absorbance (A) at λ = 286 nm versus standards ETO concentrations (μM)(d).

Section S7: DLS Results

Figure S9: Dynamic Light Scattering Analysis of CPX **5**

References

D4BnA

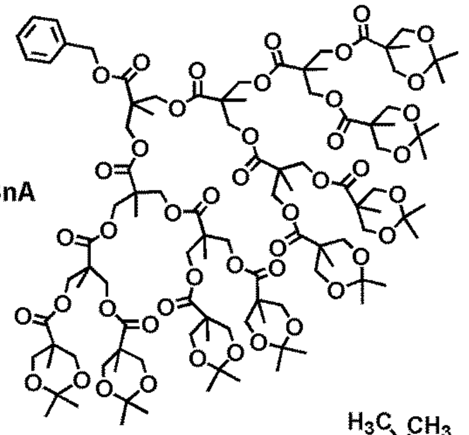

D4BnOH

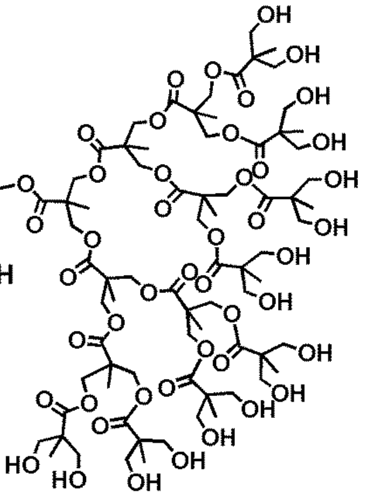

D5BnA

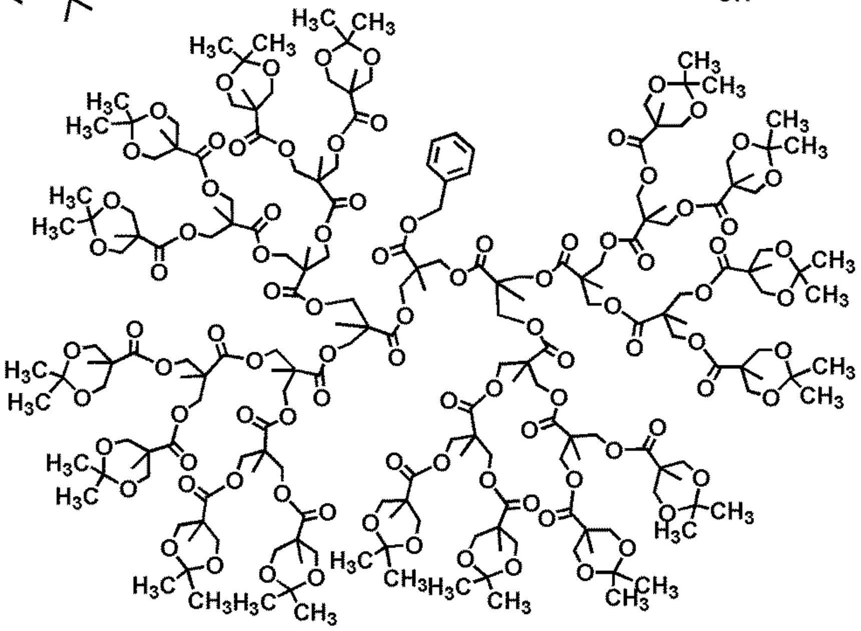

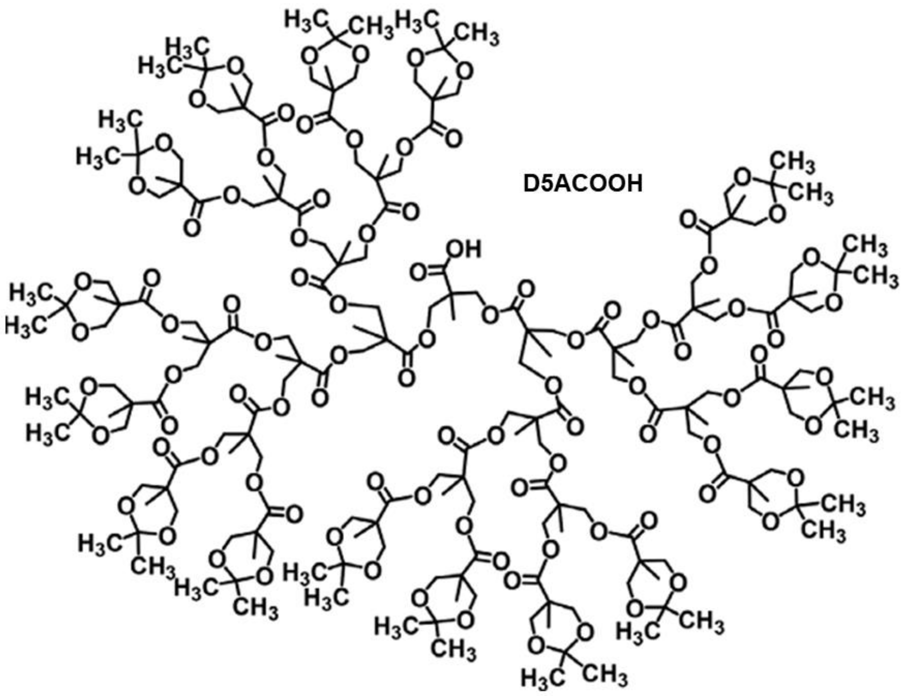

Figure S1. Structure of dendron intermediates achieved to synthetize **4**: D4BnA, D4BnOH, D5BnA and D5ACOOH

.

Section S1

Characterization data of dendrimer 4

FT-IR, NMR spectra data and Elemental analysis results of compound 4 [1]

*Dendrimer **4***. FTIR (KBr, cm^{-1}): 3433 (OH), 2933, 1733 (C=OO). ^{1}H NMR (300 MHz, DMSO-d_6), δ (ppm): 1.01, 1.16, 1.18, 1.23, 1.34 (five s signals, 186H, CH_3 of generations), 1.70 (m, 2H, CH_2 propandiol), 3.52 (dd, 128H, CH_2OH), 3.56 (partially overlapped signal, 2H, CH_2O propandiol), 3.98 (partially overlapped signal, 2H, CH_2O propandiol), 4.08-4.18 (m, 120H, CH_2O of four generations), 4.37 (br s, 64H, OH). ^{13}C NMR (75.5 MHz, DMSO-d_6) δ (ppm): 173.94, 171.73 (C=O), 64.27, 63.55 (CH_2O), 50.13 (quaternary C of fifth generation), 46.12 (other generation detectable quaternary C), 17.05, 16.61 (CH_3 of generations). Found: C, 51.71; H, 7.01. $C_{313}H_{504}O_{188}$ requires C, 51.67; H, 6.98%.

Table S1. Elemental Analysis and other physicochemical data of dendrimer **4** [1].

Compound	Formula	MW	Required (%)	Found (%)	Error (%)	Physical state
**4**	$C_{313}H_{504}O_{188}$ [1]	7275.24 [1]	C 51.67<br>H 6.98	C 51.71<br>H 7.01	C 0.04<br>H 0.03	Fluffy white hygroscopic solid

[1] Formula and MW of dendrimer **4** were estimated by ^{1}H NMR spectra and were confirmed by Elemental Analysis.

Copies of FT-IR and NMR spectra of dendrimer 4 [1]

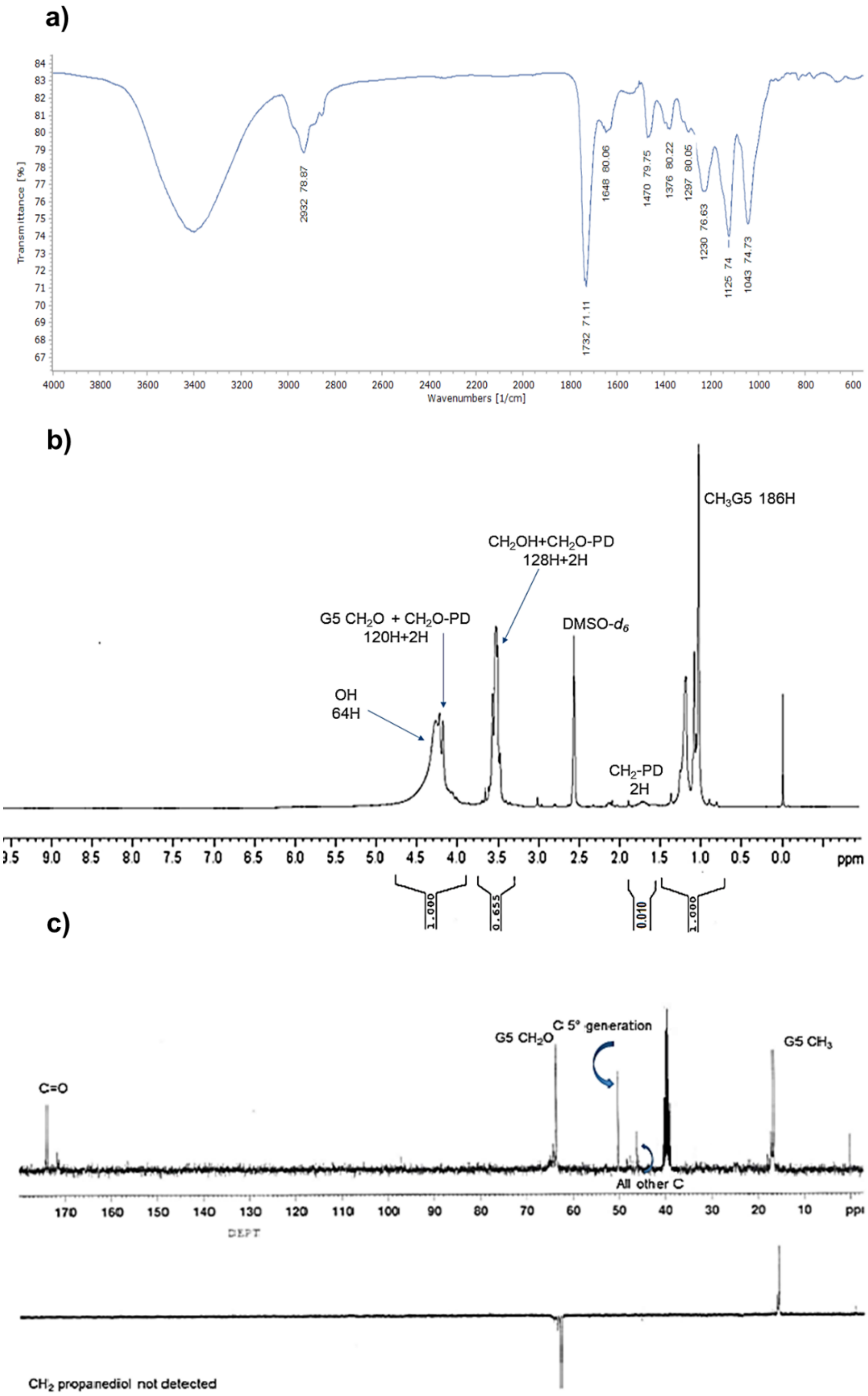

Figure S2. FTIR spectrum (KBr) (**a**), ^{1}H NMR spectrum (DMSO-*d6*, 300 MHz) (**b**) and ^{13}C NMR and DEPT 135 spectra (DMSO-*d6*, 75.5 MHz) (**c**) of dendrimer **4.**

Section S2

FTIR and NMR spectra of etoposide (ETO)

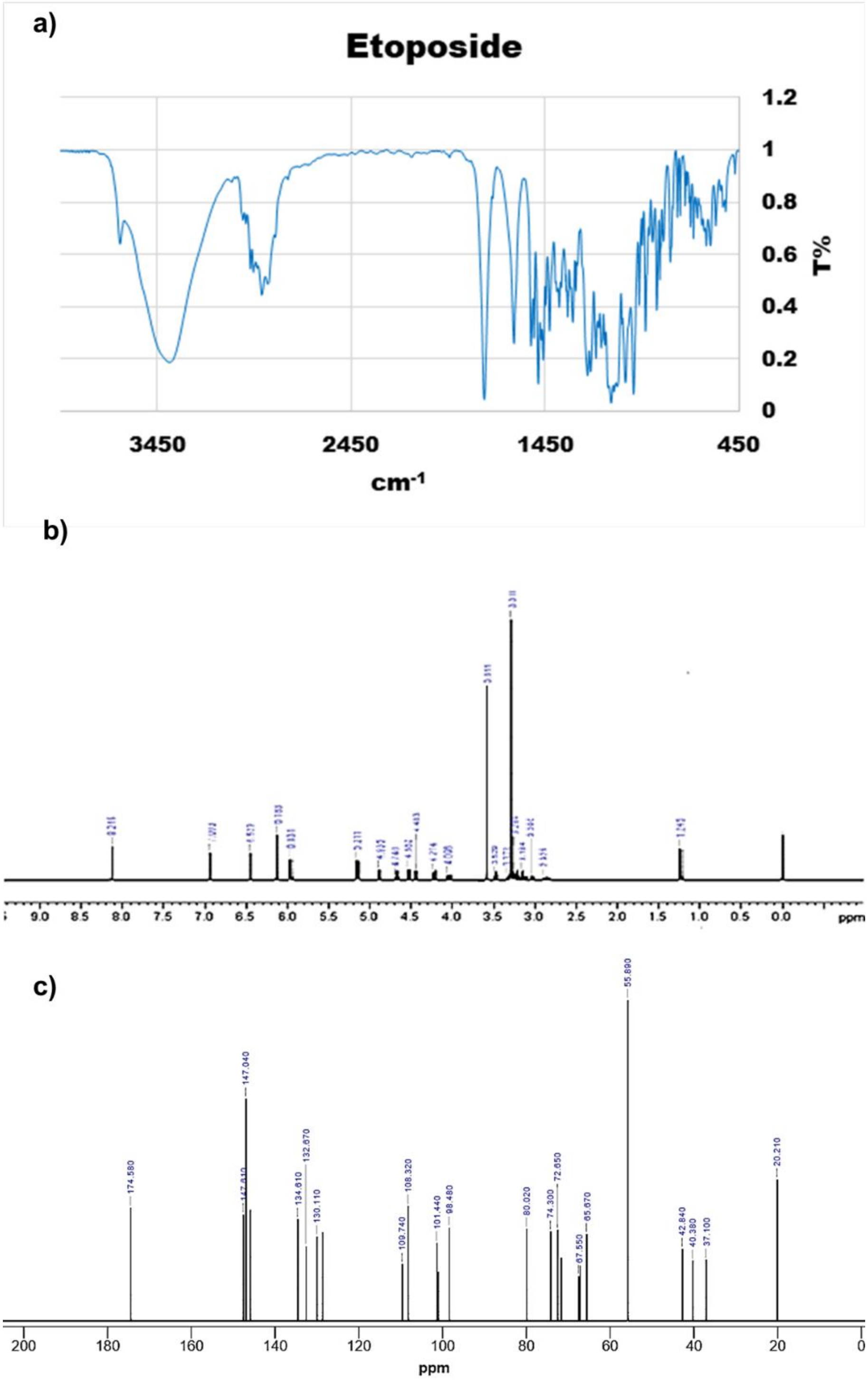

Figure S3. FTIR spectrum (KBr) (**a**), ^{1}H NMR spectrum (DMSO-*d6*, 400 MHz) (**b**) [2] and ^{13}C NMR spectrum (DMSO-*d6*, 100 MHz) (**c**) of ETO [2].

Section S3

FTIR and NMR spectra of CPX 5

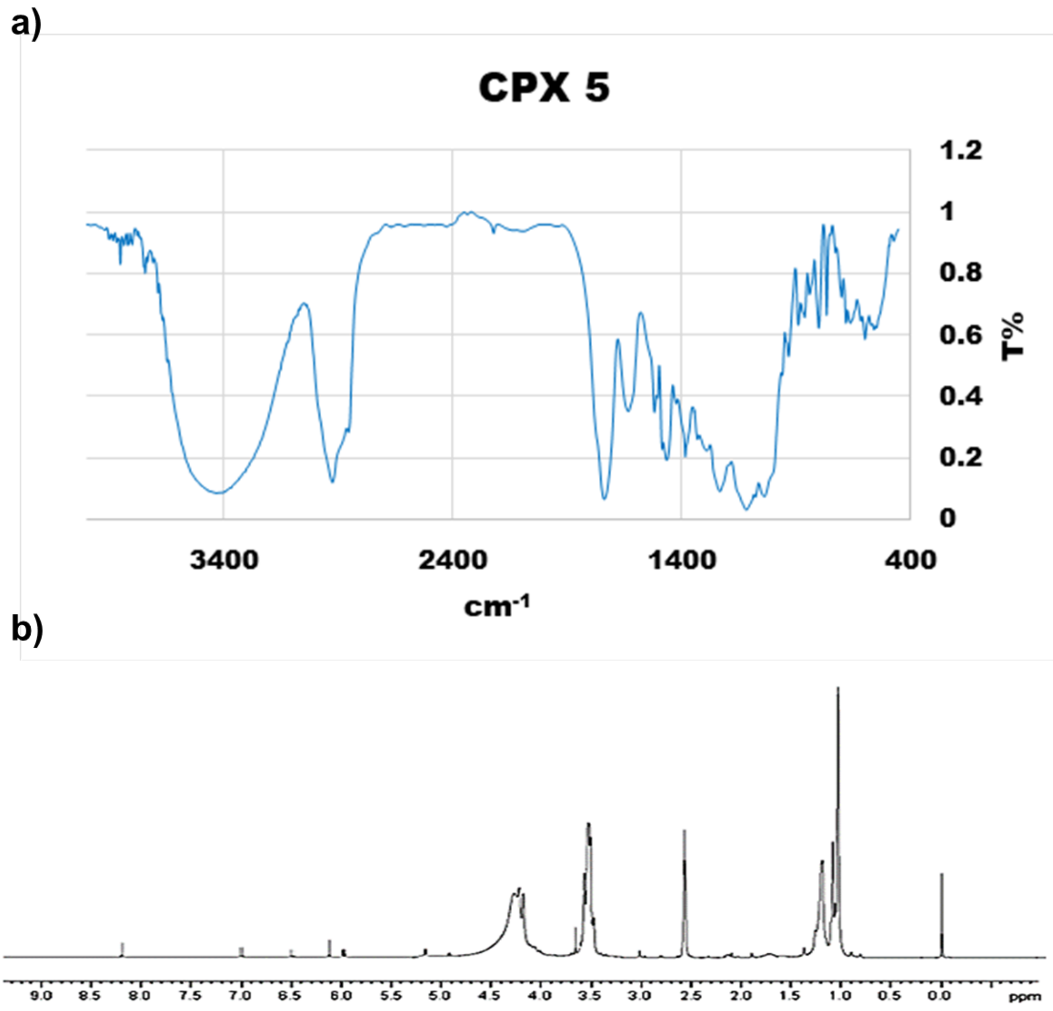

Figure S4. FTIR spectrum (KBr) (**a**) and ^{1}H NMR spectrum (DMSO-*d6*, 300 MHz) (**b**) of CPX **5**.

.

Section S4

Comparison between FTIR and NMR spectra of ETO, 4 and CPX 5

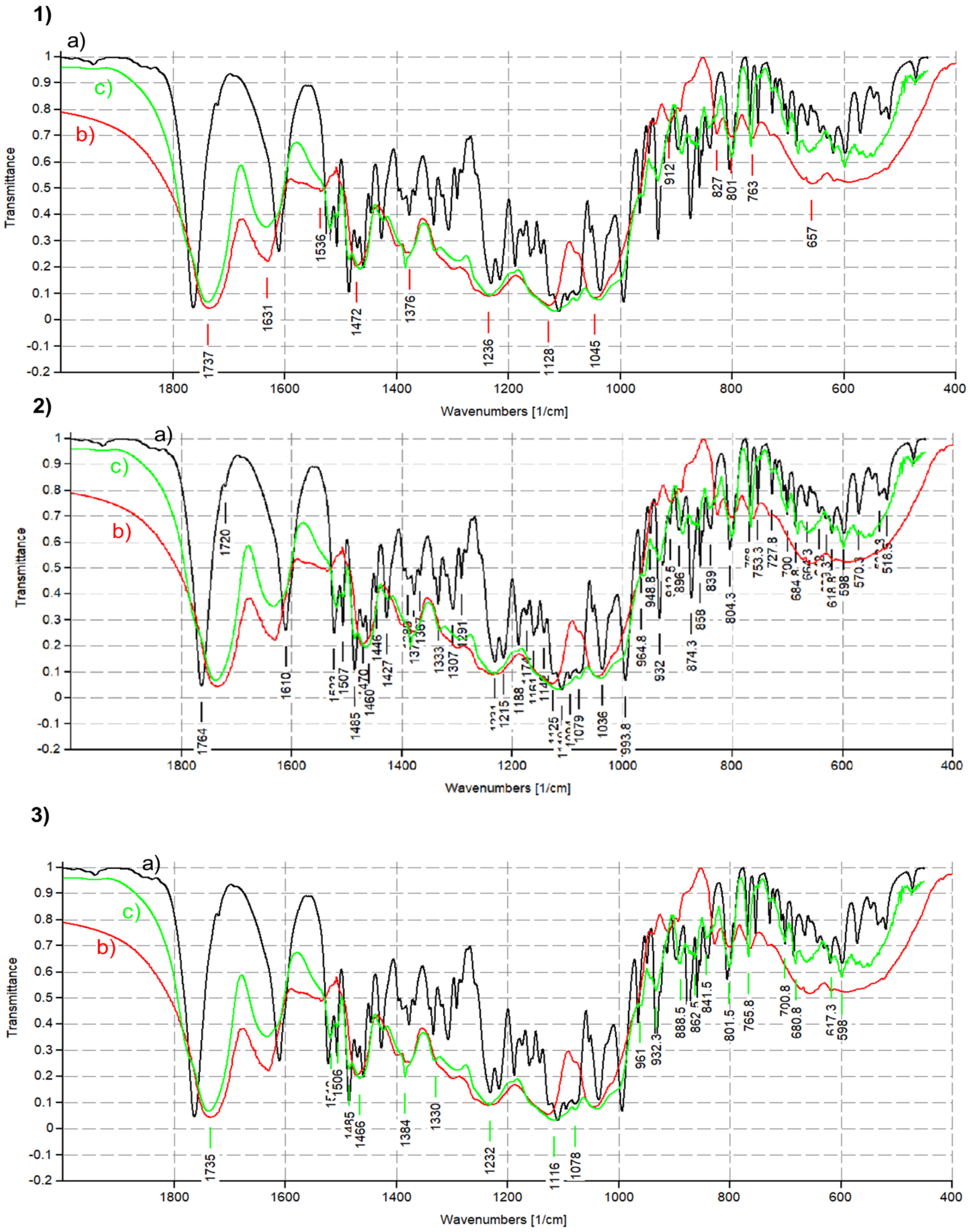

Figure S5. FTIR spectra of ETO (**a**), dendrimer **4** (**b**) and CPX **5** (**c**) with in evidence peaks of **4** (**1**), peaks of ETO (**2**) and peaks of **5** (**3**).

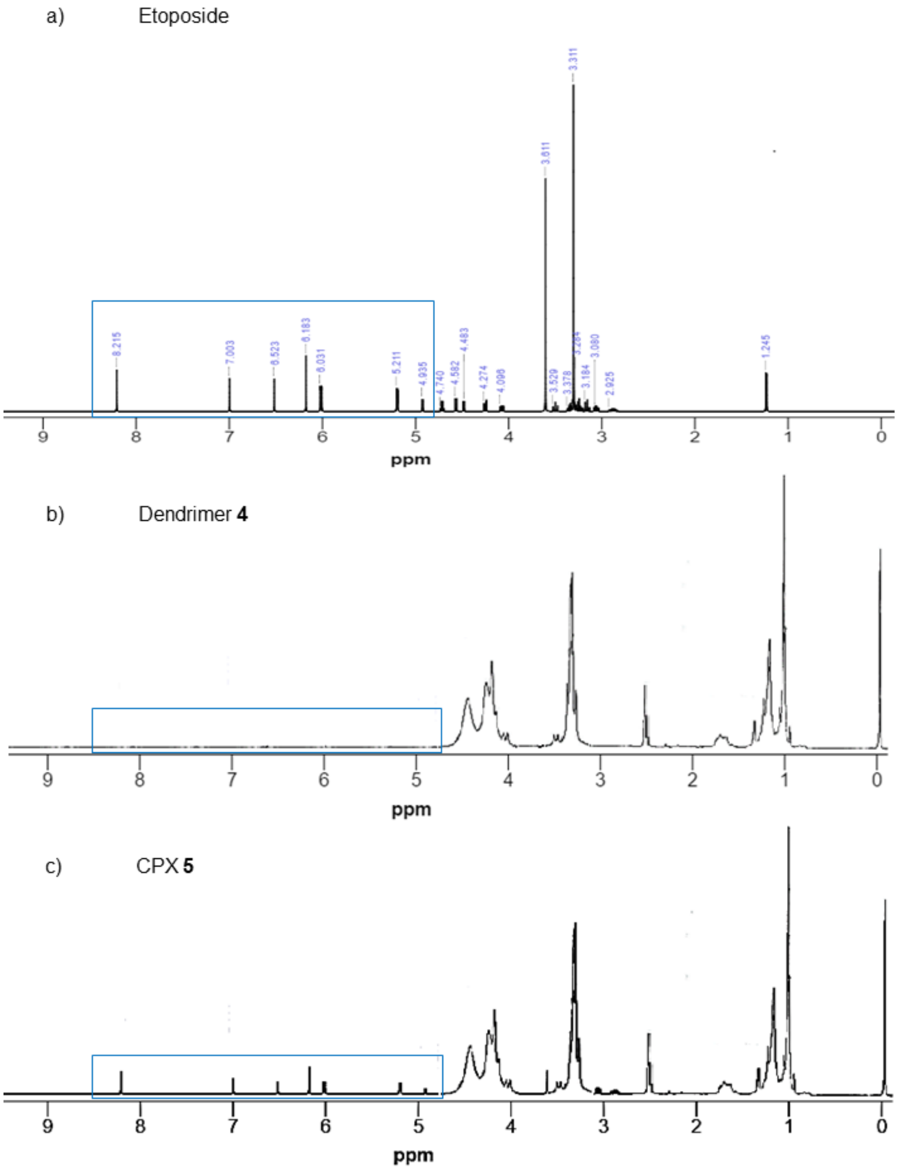

Figure S6. ^{1}H NMR spectra (DMSO-*d6*) of (**a**) ETO (400 MHz) [2], (**b**) dendrimer **4** (300MHz) and (**c**) CPX **5** (300 MHz).

Section S5

Principal Component Analysis results

PCA is a chemometric tool extensively used to process FTIR spectral data obtained from very numerous samples population to put in comparison. In PCA, multi-dimensional data are reduced to a small number of new variables – principal components (PCs) – which are orthogonal linear combinations of the original ones that efficiently represent data variability in low dimensions [3]. Information carried out by PCs is expressed in terms of percentage of explained variance. By definition, PC1 has the largest % explained variance, followed by PC2, PC3 and so on [4]. Briefly, PCA is able to put in evidence similarities or differences among the samples under study by clustering or separating them within a square of two Components identified for being Principal Components (PC).

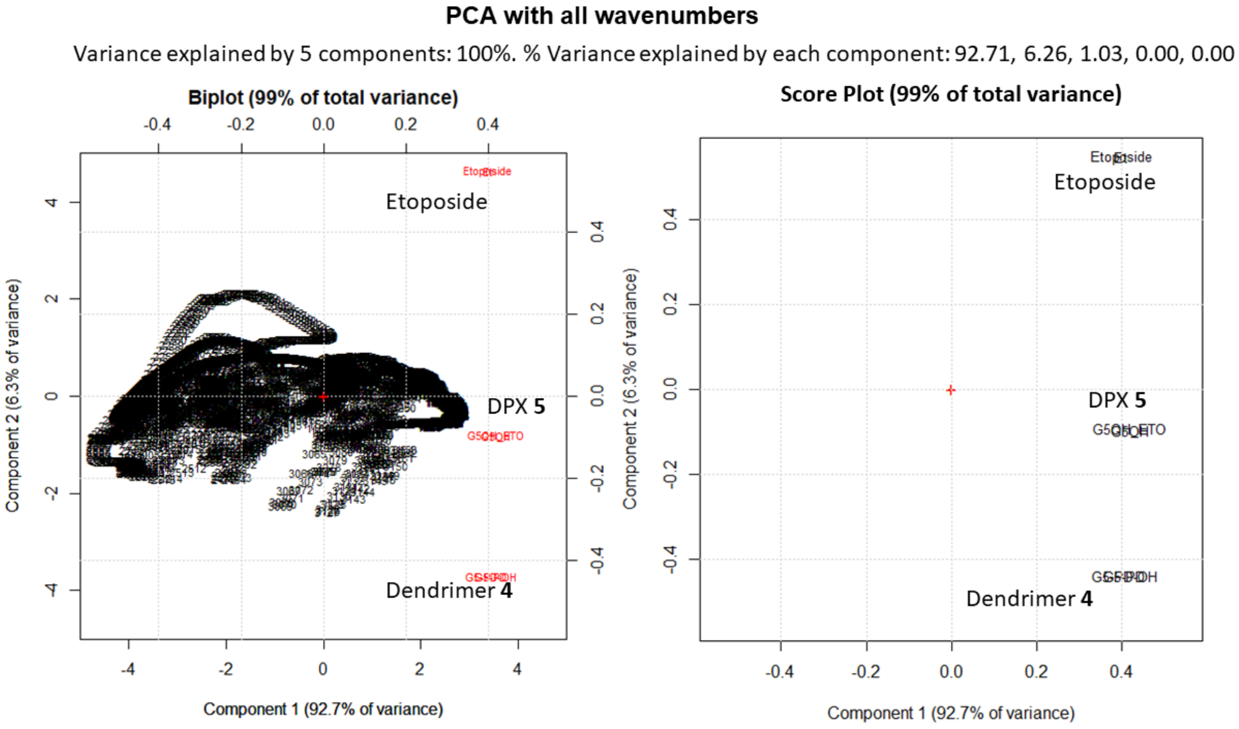

Figure S7. Bi-plot and score plot on Components PC1 and PC2.

Section S6

UV Spectrophotometric Analysis results

Table S2. Data of the calibration curve: $A_{average}$ and ETO standards μg/mL concentrations. ETO predicted concentrations (C_{ETOp}), residuals and ETO μM concentrations

C_{ETO} (μg/mL)	$A_{average}$ ± SD	C_{ETOp} (μg/mL)	Residuals	C_{ETO} (μM)
5.0	0.035 ± 0.020	4.8	-0.2	8.49
10.0	0.079 ± 0.013	10.1	+0.1	16.99
20.0	0.159 ± 0.018	19.9	-0.1	33.98
30.0	0.242 ± 0.024	30.0	0	50.97
40.0	0.321 ± 0.020	39.6	-0.4	67.96

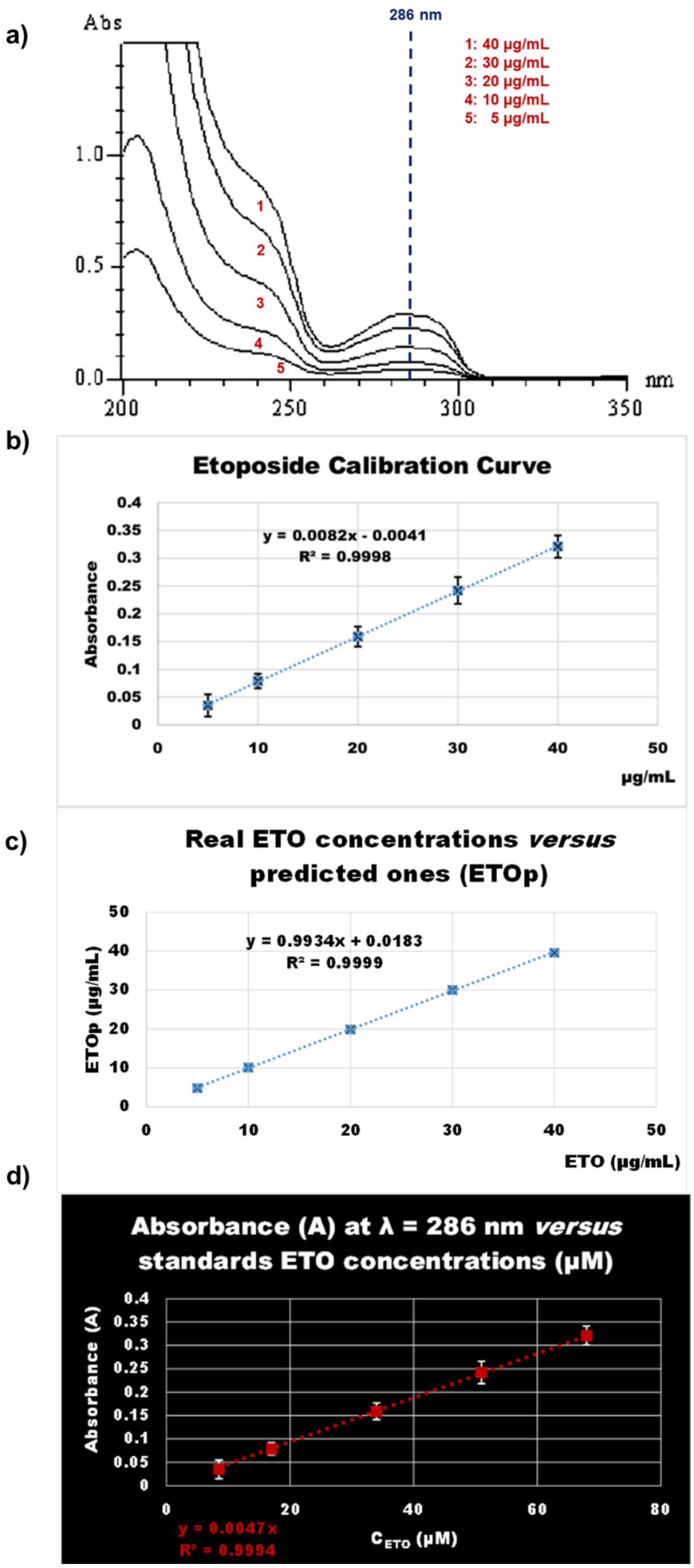

Figure S8. UV spectra of ETO dissolved in ACN/TDW 50:50 at the different concentrations used to build up the standard ETO calibration curve (**a**), standard ETO calibration curve (**b**), real ETO concentrations *versus* predicted ones (**c**), Absorbance (A) at λ = 286 nm *versus* standards ETO concentrations (µM) (**d**).

Section S7

Dynamic Light Scattering Analysis

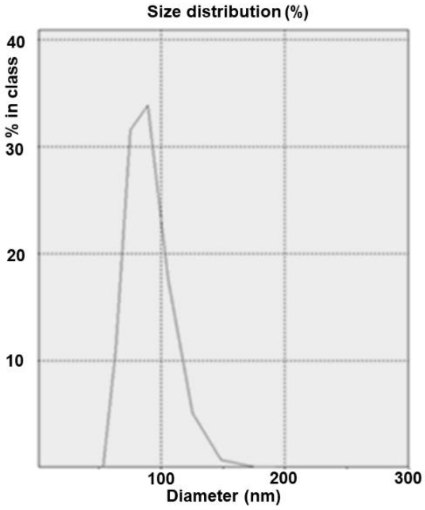

Size(nm)	Intensity	Volume	Number
13.1	0.0	0.0	0.0
16.5	0.0	0.0	0.0
20.8	0.0	0.0	0.0
26.2	0.0	0.0	0.0
32.9	0.0	0.0	0.0
41.5	0.0	0.0	0.0
52.2	0.0	9.3	22.9
65.7	16.5	20.2	47.7
82.8	5.6	12.6	26.9
104.2	0.0	1.6	2.0
131.2	0.0	0.2	0.0
165.2	4.3	1.0	0.1
208.0	18.5	2.0	0.2
261.8	19.3	2.2	0.1
329.7	7.3	1.3	0.0
415.1	0.0	0.4	0.0
522.6	0.0	0.0	0.0
658.0	0.0	0.2	0.0
828.4	2.7	5.1	0.0
1043.0	8.8	14.2	0.0
1313.2	10.8	16.9	0.0
1653.4	6.1	10.1	0.0
2081.7	0.1	2.6	0.0
2621.0	0.0	0.0	0.0

Figure S9. Dynamic Light Scattering Analysis of CPX **5**

References

[1] Alfei, S.; Catena, S.; Turrini, F. Biodegradable and biocompatible spherical dendrimer nanoparticles with a gallic acid shell, as double-acting, high performant antioxidant device to fight diseases from "oxidative stress", *Drug Deliv. Transl. Res.* **2019**. https://doi.org/10.1007/s13346-019-00681-8.

[2] Spectral Database for Organic Compounds (SDBS): https://sdbs.db.aist.go.jp/sdbs/cgi-bin/direct_frame_top.cgi (latest access, 24 November 2019).

[3] Jolliffe, I.T.; Cadima, J. Principal component analysis: a review and recent developments. *Philos. Trans. A Math. Phys. Eng. Sci.* **2016**, *374*, 20150202.

[4] Alfei, S.; Oliveri, P.; Malegori, C. Assessment of the Efficiency of a Nanospherical Gallic Acid Dendrimer for Long-Term Preservation of Essential Oils: An Integrated Chemometric-Assisted FTIR Study. *ChemistrySelect* **2019**, *4*, 8891 -8901.
